# Hypertension management and health belief in middle-aged laotian population: a cross-sectional survey

**DOI:** 10.1186/s12889-025-24638-4

**Published:** 2025-10-03

**Authors:** Younhee Kang, Boeun Kim

**Affiliations:** 1 Division of Nursing, College of Nursing, Graduate Program in System Health Science and Engineering, Ewha Womans University, Seoul, Republic of Korea; 2 University of Iowa College of Nursing, 50 Newton Road, Iowa City, IA 52242 USA

**Keywords:** Hypertension, Health beliefs, Surveys, Underserved populations

## Abstract

**Background:**

Despite the growing burden of cardiovascular diseases in the Lao People’s Democratic Republic (Lao PDR), studies on hypertension—a key modifiable risk factor—remain limited. There is an urgent need to understand regional differences in hypertension prevalence and its management, particularly across rural and urban settings. Health locus of control (HLOC), reflecting individuals’ beliefs about health determinants, may influence hypertension awareness, treatment, and control. This study estimated the prevalence and management of hypertension and examined their associations with HLOC among middle-aged adults in Lao PDR by residential area.

**Methods:**

A cross-sectional, population-based survey was conducted among adults aged 40 to 59 years living in Vientiane Capital and Vientiane Province using a multistage cluster sampling approach. The analytic sample size for estimating hypertension prevalence included 922 participants, while 441 participants with hypertension (urban: *n* = 230; rural: *n* = 211) were included in the association analysis. Blood pressure was measured using a digital automatic blood pressure monitor. Hypertension awareness and treatment were assessed through self-report, and control was defined based on measured blood pressure. HLOC was measured using the Multidimensional Health Locus of Control Scale (internal, chance, doctor, and other people subscales). The Horvitz-Thomson estimator was used to calculate prevalence, and weighted logistic regression models were used to examine associations.

**Results:**

Of the 922 participants, 52.2% were aged 40–49 years, 55.4% were female, and 86.6% identified as Lao-Tai, the majority ethnic group. Among those with hypertension, weighted percentages of awareness, treatment, and control were 43.4%, 42.3%, and 17.8%, respectively. In urban areas, higher doctor and other people HLOC scores were associated with greater odds of awareness, treatment, and control. In rural areas, higher internal HLOC score was associated with greater odds of hypertension control.

**Conclusions:**

The findings highlight the need for targeted actions to prevent and manage hypertension in Lao PDR. Health beliefs—including differences in health locus of control between urban and rural populations—should be considered in the development of culturally tailored interventions to improve hypertension outcomes.

**Supplementary Information:**

The online version contains supplementary material available at 10.1186/s12889-025-24638-4.

## Background

Non-communicable diseases, including cancer, ischemic heart disease, cirrhosis, and dementia account for 39.8 million deaths worldwide [1]. Cardiovascular disease is the second leading cause of death globally [2]. The burden of cardiovascular disease is disproportionately rising in low- and middle-income countries [3]. Hypertension is a major risk factor for cardiovascular disease, and it can be prevented or managed by lifestyle modifications [4, 5]. Preventing and controlling hypertension may reduce the mortality and morbidity from cardiovascular disease [5]. However, the prevalence of hypertension has been increased in low- and middle-income countries [6], where access to preventive care and treatment is often limited. This growing trend highlights the urgent need for targeted public health interventions and policies to address hypertension in these settings.

The Lao People’s Democratic Republic (Lao PDR) is one of the low- and middle-income countries (LMICs) in Southeast Asia, with a population of approximately 8 million [7, 8]. In Laos PDR, 60% of all deaths are due to non-communicable diseases, and cardiovascular disease contributes to nearly half of the deaths [9]. However, studies on hypertension—a key modifiable risk factor for cardiovascular disease— among Laotians are limited, hindering the development of effective strategies to reduce the cardiovascular disease burden. A study conducted in 2008 in one municipality estimated the prevalence of hypertension and found that 22.3% of adults aged 25 to 64 years had hypertension, while 83.8% of those with hypertension were not taking medication [9]. Another study, using a nationally representative sample of adults aged 18 to 64 years, was conducted in 2013 to estimate the prevalence of hypertension, as well as the proportions of individuals who were aware of their condition, were receiving treatment, and had controlled blood pressure [10]. This study found that one-fifth of participants had hypertension, and among those, 29.3% were aware of their condition, 18.2% were receiving treatment, and 16.7% had controlled blood pressure [10]. There remains a gap in understanding how hypertension prevalence and management vary across geographic settings within the country.

Lao PDR is home to over 50 ethnic groups who speak more than 80 distinct languages, many of whom live in remote areas that are difficulty to access [7, 11]. Significant disparities in healthcare access exist between urban and rural areas, with rural areas having fewer resources. These disparities are driven by multiple factors, including limited availability of healthcare facilities, poor transportation infrastructure, shortages of healthcare providers, frequent stockouts of essential medicines, lack of health information, and a higher proportion of ethnic minorities who may face language barriers and live under the poverty line [7]. These barriers can significantly affect individuals’ awareness of hypertension and their ability to manage the condition, yet no studies have examined how these outcomes differ between rural and urban populations.

Health locus of control may be another important factor influencing hypertension prevention and management in the context of Lao PDR. Health locus of control refers to individual beliefs in relation to control of one’s health [12]. Four types of health locus of control have been identified: internal—the belief that one’s health is determined by personal behaviors; chance—the belief that health is influenced by fate or luck; doctor—the belief that health is primarily affected by a doctor; and other people—the belief that health is influenced by significant individuals such as family members or friends [13]. Health locus of control can shape health behaviors, adherence to treatment, or willingness to engage in an intervention [13–15], all of which are closely related to the prevention and management of hypertension. Notably, in Lao PDR, cultural and traditional beliefs, along with family norms and community dynamics, play a significant role in sharing individuals’ engagement with healthcare services [7, 16, 17]. For example, more than 90% of deaths and about 30% of births occur outside of health facilities [7]. Given these dynamics, individuals’ beliefs about who or what controls their health may play a pivotal role in how they respond to health information, seek care, and adhere to treatment for hypertension in Lao PDR. However, there is limited literature on how health locus of control is related to hypertension awareness, receiving treatment, and control among Laotians.

Accordingly, this study estimated the prevalence of hypertension, along with proportions of individuals aware of their conditions, receiving treatment, and had controlled blood pressure, among adults aged 40 to 59 years living in urban and rural areas of Vientiane, Lao PDR. This age range was selected because the risk of mortality related to vascular diseases, including hypertension, begins to increase significantly around the age of 40 years [18]. In addition, the life expectancy in Lao PDR at the time of data collection (2015) was 66.9 years [19]. Given this context, adults aged 40 to 59 years represent a key segment of the population entering a high-risk period for hypertension-related morbidity. Early detection and management in this age group are therefore critical for reducing preventable disease burden and premature mortality. This study further examined the associations of each type of health locus of control with hypertension awareness, treatment, and control status. We hypothesized that doctor health locus of control would be associated with greater hypertension awareness, treatment, and control in urban setting, whereas internal health locus of control would be associated with greater awareness, treatment, and control in rural setting.

## Methods

### Study design

A cross-sectional study was conducted to implement a population-based survey in Lao PDR. Data collection, including blood pressure measurement and questionnaire administration, was carried out by trained research staff using a standardized protocol. Two data collection teams were formed, each consisting of seven members: one team leader, five interviewers, and two research assistants who were healthcare professionals or students in health-related fields in Lao PDR. All team members received training prior to data collection. We administered the questionnaire at participants’ homes to ensure confidentiality, and blood pressure was measured at a community center or temple within the village. Given the high illiteracy rate, research staff were available to assist participants in completing the survey. A research coordinator from South Korea oversaw the data collection process, providing supervision and offering resources as needed. Data collection was conducted from May 2 to June 5, 2015. This study was approved by Institutional Review Boards in South Korea and Lao PDR (89 − 12).

### Study participants

The target population was individuals aged 40 to 59 years residing in either Vientiane or Vientiane Province, Lao PDR. Lao PDR is administratively divided into 17 provinces, including Vientiane Province, and one capital city municipality, Vientiane. Individuals were eligible to participate if they were aged 40 to 59 years and resided these areas. Individuals with critical illnesses or those who could not speak or understand Lao were excluded, as this could have affected their ability to participate.

**Fig. 1:**
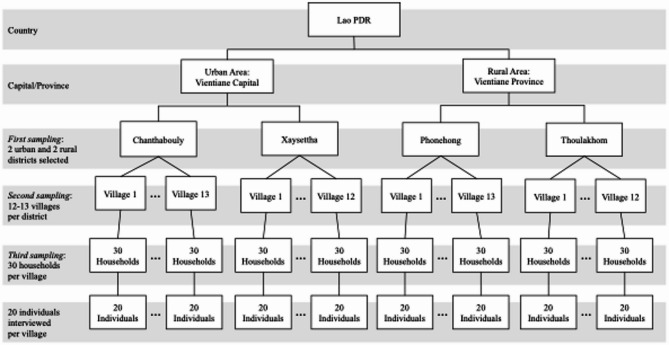
A multistage cluster sampling process in Lao People’s Democratic Republic (Lao PDR)

The sampling design was a multistage cluster sampling (Fig. 1). In the first stage, we selected four representative districts (cluster)—two from Vientiane to represent urban areas (Chanthabouly District, Xaysettha District, selected from a total of nine districts) and two from Vientiane Province to represent rural areas (Phonehong District, Thoulakhom District, selected from a total of ten districts)—in terms of population size and socioeconomic characteristics. In the second stage, 12 or 13 villages were randomly selected from each district, totaling 50 villages—25 for urban areas and 25 for rural areas. In the third stage, we contacted the village head to obtain approval to conduct the survey and to request a list of all households in the village. In each village, a random starting number was drawn, and subsequent households were selected by consistently adding that number to identify the next household in the sampling frame. To account for potential refusals or non-availability at the time of data collection, 30 households were initially selected per village, with the goal of completing 20 interviews in each. Finally, within the selected households, one eligible individual was interviewed. If multiple eligible individuals were present in a household, one was selected arbitrarily based on their convenience and willingness to participate. If no eligible individuals were present in a selected household, the household was skipped. The target sample size of 1000 participants was determined to estimate the prevalence of chronic diseases, including hypertension, in the target population. As this is a secondary data analysis, the original sample size was not calculated based on the specific outcomes examined in this study. However, a post hoc power analysis indicated that a minimum sample of 211 participants would be sufficient to detect a small effect size (Odds Ratio [OR] = 1.5) with 80% power at a 5% significance level (two-sided). The required sample size was estimated using G*Power software (version 3.1.9).

### Blood pressure measurement

Participants rested for at least 15 min and refrained from smoking for at least 30 min before blood pressure measurement. Participants laid their arm on a table and the middle of the cuff positioned at the level of the midpoint of the sternum. Sitting blood pressure was measured once in each arm by trained research assistants using a digital automatic blood pressure monitor (Accumed AW150f), with the two measurements taken 3 min apart. The arm with the higher systolic blood pressure reading was identified, and both the systolic and diastolic readings from that arm were used for the analysis. This approach was chosen because measurements from the arm with higher values have been shown to better predict all-cause mortality, cardiovascular mortality, and cardiovascular events than those from the arm with lower values [20, 21]. Hypertension was defined as self-reported diagnosis of hypertension with current use of blood pressure-lowering medication, or a measured systolic blood pressure greater than 140 mmHg and/or diastolic blood pressure greater than 90 mmHg [20, 22].

### Primary outcomes: hypertension awareness, treatment, and control

The primary outcomes were awareness of having hypertension, receipt of treatment, and blood pressure control. Among participants identified as having hypertension, awareness of having hypertension was defined as having ever received a medical diagnosis of hypertension by a healthcare provider. Treatment was defined as current use of blood pressure-lowering medication. Controlled status of blood pressure was defined as having a systolic blood pressure below 140 mmHg and/or a diastolic blood pressure below 90 mmHg [22].

### Exposures: health locus of control

The exposure was health locus of control. Health locus of control was measured using Multidimensional Health Locus of Control Scales form C (MHLC) [13]. The MHLC consists of 18 items across four subscales, including internal (6 items), chance (6 items), doctors (3 items), and other people (3 items). Example items from each subscale include statements about the role of personal behavior (internal), external forces such as fate or luck (chance), medical professionals (doctors), or social influences (other people). Responses were rated on 6-point Likert scale ranging from 1 (strongly disagree) to 6 (strongly agree), with higher scores indicating greater agreement with the respective belief domain. Internal consistency in this study sample was fair to good, with Cronbach’s α values of 0.82, 0.78, 0.73, and 0.73 for the internal, chance, doctor, and other people subscales, respectively.

### Covariates

Sociodemographic factors and other health related factors were asked, including age, gender, marital status, ethnicity, employment status, educational attainment, literacy, health insurance status, residence (urban or rural, based on Lao Statistical Bureau), high-risk drinking behaviors, and smoking status. High-risk drinking was defined as consumption of 4 or more drinks on any day in the previous 30 days for women and 5 or more drinks on any day in the previous 30 days for men [23]. Ever smokers were defined as individuals who had smoked at least 100 cigarettes during their lifetime [24]. Literacy was assessed through a self-reported question asking participants whether they could read Lao.

### Data analysis

Based on the sampling design, selection propensity was computed as the product of probability at each sample selection stage. The missing rate was 5.8% (*n* = 58), and the sampling weight was adjusted for missing data using propensity stratification [25]. Design weight was calculated as the inverse final selection probability [25].

The Horvitz-Thomson estimator was used to calculate overall prevalence of hypertension among all participants, as well as the proportions of hypertension awareness, treatment and control among participants identified as hypertensive. Weighted logistic regression models, accounting for clustering of participants within villages, were used to examine the associations between each health locus of control subscale and hypertension awareness, treatment, and control. Each health locus of control subscale was analyzed separately in relation to the outcomes, stratified by residential area (rural or urban). Each model was adjusted for age, gender, level of education attainment, employment status, ethnicity, and insurance status. The odds ratios (OR) along with 95% confidence intervals (CI) were reported, with statistical significance set at *p* < 0.05 (two-sided). All analyses were conducted using R statistical software [26].

## Results

A total of 996 individuals were approached and agreed to participate; however, 16 were found to be ineligible due to not meeting the age criteria. Out of the 980 eligible survey participants, 58 subjects had missing item responses. A total of 922 participants were included in this analysis (Table 1). Among them, 52.2% were aged 40 to 49 years, and 55.4% female. The majority of participants were married (86.8%), identified as ethnically Lao-Tai (86.6%), and employed (82.4%). A total of 17.9% of participant had no formal education or had attended less than primary school, and 11.8% were illiterate. Overall, only 25.0% of participants had any kind of health insurance, with coverage significantly lower among rural residents (16.6%) than urban residents (33.8%). More than half of the participants (58.1%) were classified as high-risk drinkers, and 26.8% had ever smoked. Individuals living in urban areas, compared to those in rural areas, were more likely to be female, belong to the major ethnic group (i.e., Lao-Tai), have attained at least a tertiary-level education, be literate, and have health insurance. Additionally, urban participants had higher internal and doctor health locus of control scores, while rural participants had higher chance health locus of control scores (Table 1).

**Table 1:** Unweighted characteristics of participants by residential area

Characteristics	*n* (%)		
	Rural (*n* = 469)	Urban (*n* = 453)	Total (*N* = 922)
Age			
40–49	244 (52.0)	237 (52.3)	481 (52.2)
50–59	225 (48.0)	216 (47.7)	441 (47.8)
Gender			
Male	232 (49.5)	179 (39.5)	411 (44.6)
Female	237 (50.5)	274 (60.5)	511 (55.4)
Marital status			
Never married	6 (1.3)	18 (4.0)	24 (2.6)
Married or cohabiting	406 (86.6)	394 (87.0)	800 (86.8)
Separated/divorced/widowed	57 (12.2)	41 (9.0)	98 (10.6)
Ethnicity			
Lao-Tai	349 (74.4)	449 (99.1)	798 (86.6)
Other	120 (25.6)	4 (0.9)	124 (13.4)
Employment status			
Employed/paid job	422 (90.0)	338 (74.6)	760 (82.4)
Not employed/non-paid job	47 (10.0)	115 (25.4)	162 (17.6)
Educational attainment			
No formal/less than primary	130 (27.7)	35 (7.7)	165 (17.9)
Primary	145 (30.9)	87 (19.2)	232 (25.2)
Secondary	102 (21.8)	101 (22.3)	203 (22.0)
Tertiary or more	92 (19.6)	230 (50.8)	322 (34.9)
Literacy			
Yes	383 (81.7)	430 (94.9)	813 (88.2)
No	86 (18.3)	23 (5.1)	109 (11.8)
Health insurance			
Yes	78 (16.6)	153 (33.8)	231 (25.0)
No	391 (83.4)	300 (66.2)	691 (75.0)
High-risk drinking			
Yes	245 (52.2)	291 (64.2)	536 (58.1)
No	224 (47.8)	162 (35.8)	386 (41.9)
Smoker			
Ever smoked	137 (29.2)	110 (24.3)	247 (26.8)
Never smoked	332 (70.8)	343 (75.7)	675 (73.2)
Health locus of control			
Internal, mean (SD)	27.6 (4.6)	28.8 (5.2)	28.2 (4.9)
Chance, mean (SD)	24.6 (5.4)	23.1 (6.5)	23.8 (6.0)
Doctor, mean (SD)	14.8 (2.1)	15.4 (2.2)	15.1 (2.2)
Other, mean (SD)	13.1 (2.9)	13.0 (3.3)	13.0 (3.1)

### Weighted proportions of hypertension, awareness, treatment, and control

The weighted prevalence of hypertension among participants was 44.3% (Table 2). The prevalence of hypertension was higher among individuals living in urban areas, as compared to those living in rural areas (46.0% and 41.6% respectively). Among individuals with identified hypertension (*n* = 441), 43.4% were aware of their hypertension condition, and 42.3% were taking blood pressure-lowering medications. Blood pressure was controlled in only 17.8% of the individuals with hypertension. A greater proportion of individuals living in rural areas were aware of their hypertension and reported receiving treatment; however, the proportion of individuals with controlled hypertension was higher in urban areas.

**Table 2:** Weighted prevalence of hypertension, and proportions of awareness, treatment, and control by residential area

	*% *(*SE*)			
	Hypertension (*N* = 922)	Awareness (*n* = 441)	Treatment (*n*= 441)	Control (*n* = 441)
Overall	44.3 (2.2)	43.4 (3.1)	42.3 (3.2)	17.8 (2.2)
Residence				
Urban	46.0 (3.2)	42.9 (4.3)	41.6 (4.4)	18.3 (2.8)
Rural	41.6 (2.4)	44.2 (4.1)	43.7 (3.9)	17.0 (3.4)

### Association between health locus of control and hypertension awareness, treatment, and control among participants living in urban areas

Among participants with identified hypertension status, higher scores on the doctor and other health locus of control subscales were associated with greater levels of hypertension awareness, treatment, and control among those living in urban areas (Table 3). Individuals with one point greater in doctor health locus of control were 15% more likely to be aware of their hypertension status (95% CI: 1.03–1.28, *p* = 0.022), 13% more likely to take blood pressure-lowering medications (95% CI: 1.01–1.27, *p* = 0.046), and 25% more likely to have controlled hypertension (95% CI: 1.11–1.41, *p* = 0.002). Individuals with one point higher in other health locus of control were 16% more likely to be aware of their hypertension status (95% CI: 1.06–1.27, *p* = 0.005), 14% more likely to receive blood pressure-lowering medications (95% CI: 1.04–1.26, *p* = 0.016), and 14% more likely to have controlled hypertension (95% CI: 1.02–1.28, *p* = 0.038). Internal and chance health locus of control subscale scores were not associated with hypertension awareness, treatment, or control among urban residents with hypertension.

**Table 3:** Weighted association between health locus of control and hypertension awareness, treatment, and control among urban participants (n = 230)

		*OR* (95% CI)	
	Awareness	Treatment	Control
Internal HLOC	1.05 (1.00-1.11)	1.05 (1.00-1.10)	1.03 (0.96-1.11)
Chance HLOC	1.03 (0.98-1.08)	1.03 (0.99-1.08)	1.01 (0.94-1.08)
Doctor HLOC	**1.15 (1.03-1.28)**	**1.13 (1.01-1.27)**	**1.25 (1.11-1.41)**
Other HLOC	**1.16 (1.06-1.27)**	**1.14 (1.04-1.26)**	**1.14 (1.02-1.28)**

*HLOC *Health locus of control, *OR *Odds ratio, *CI *Confidence internal

Separate models were fit for each combination of independent and dependent variables among individuals with hypertension living in urban areas. All models were adjusted for age, sex, educational attainment, employment status, ethnicity, and health insurance coverage. Analytic weights were applied to account for nonresponse and clustering of participants within villages

**Table 4:** Weighted association between health locus of control and hypertension awareness, treatment, and control among rural participants (*n* = 211)

		*OR* (95% CI)	
	Awareness	Treatment	Control
Internal HLOC	1.06 (0.98-1.15)	1.06 (0.98-1.15)	**1.17 (1.05-1.29)**
Chance HLOC	**1.06 (1.02-1.11)**	1.04 (0.98-1.12)	1.09 (0.99-1.21)
Doctor HLOC	1.14 (0.98-1.34)	1.15 (0.99-1.35)	1.07 (0.81-1.39)
Other HLOC	1.10 (0.99-1.23)	1.10 (0.99-1.23)	1.22 (1.00-1.48)

*HLOC *Health locus of control, *OR *Odds ratio, *CI *Confidence internal

Separate models were fit for each combination of independent and dependent variables among individuals with hypertension living in rural areas. Each model was adjusted for age, sex, education attainment, employment status, ethnicity, and insurance. Analytic weights were applied to account for nonresponse and clustering of participants within villages

### Association health locus of control and hypertension awareness, treatment, and control among participants living in rural areas

Internal locus of control was positively associated with controlled hypertension, but not with hypertension awareness or treatment, among rural residents with hypertension (Table 4). Individuals with one point higher in internal health locus of control was related to 17% higher likelihood of having controlled hypertension (95% CI: 1.05–1.29, *p* = 0.009). A higher score in chance health locus of control subscale score was not associated with greater odds of being aware of one’s hypertension status, receiving treatment, or having controlled blood pressure. Unlike urban participants, doctor and other health locus of control subscales were not associated with hypertension awareness, treatment, and control among rural residents with hypertension.

## Discussion

This cross-sectional survey provides estimates of prevalence of hypertension among middle-aged adults living in Vientiane Capital and Vientiane Province, Lao PDR—areas where population-level data on hypertension remain limited. Notably, the findings reveal disparities in hypertension management and control between rural and urban populations. Furthermore, the study identified distinct patterns in the associations between health locus of control and hypertension awareness, treatment, and control across rural and urban areas. These insights can help explain urban–rural disparities in hypertension outcomes and inform the development of tailored intervention strategies in this high-need context, where the burden of cardiovascular disease remains substantial but both resources and data remain limited.

In this population-based sample of 922 adults aged 40 to 59 years from Vientiane Capital and Vientiane Province, Lao PDR, surveyed in 2015, the overall prevalence of hypertension was 44.3%. Among the 441 individuals identified as having hypertension, 43.4% were aware of their condition, 42.3% reported receiving treatment, and 17.8% had controlled blood pressure. In the first national survey conducted in Lao PDR in 2013, which included 2,543 individuals aged 18 to 64 years, the prevalence of hypertension was 27.5% among those aged 45 to 54 years (*n* = 535) [10]. Among hypertensive individuals in this age group, 36.0% were aware of their condition, 21.8% received treatment, and 13.5% had controlled blood pressure [10]. The higher prevalence observed in our study may reflect differences in sample characteristics, including a specific age range and a higher proportion of participants residing in urban areas (49.1% in our survey compared to 32.3% in the national survey). Urban residents in Lao PDR are more frequently exposed to hypertension risk factors, including westernized dietary patterns such as a higher frequency of eating out and increased consumption of fatty meats and fried or stir-fried foods, as well as higher rates of obesity [27]. These factors likely contribute to the higher prevalence of hypertension [28] observed in our study. In our survey, we found a higher prevalence of hypertension in urban areas compared to rural areas (46.0% vs. 41.6%). Compared to the national survey [10], the higher levels of hypertension awareness, treatment, and blood pressure control observed in our study may be attributed, in part, to a larger share of urban residents (49.1% vs. 32.3%), where healthcare services and a more qualified health workforce are concentrated compared to rural and remote areas [29], a greater proportion of participants with secondary education or higher (56.9% vs. 30.8%), and a higher percentage identifying as Lao-Tai, the majority ethnic group (86.6% vs. 73.0%).

We found that a greater proportion of individuals living in rural areas were aware of their hypertension and reported receiving treatment; however, the proportion of individuals with controlled hypertension was higher among urban residents. In Lao PDR, each province has one provincial public hospital, and each district typically has one district hospital along with five to ten health centers [30]. In rural areas, government-managed health centers serve as the primary providers of care, while in urban areas, a growing number of private clinics and hospitals complement services delivered by government facilities [29, 31]. Diagnosing hypertension does not require advanced equipment, and access to primary care in rural areas may help facilitate awareness and initial treatment. However, several factors may hinder effective hypertension control in rural areas. These include limited access to follow-up care, potentially related to lower rates of health insurance coverage—particularly given that the survey was conducted in 2015, prior to the launch of the National Health Insurance scheme in 2016—and reduced access to transportation and consistent medication availability. In our sample, only 16.6% of rural participants reported having health insurance, compared to 33.8% of those living in urban areas. In addition to systemic barriers, individual-level factors may also contribute to lower rates of hypertension control in rural areas. A larger proportion of rural participants had no formal education or had completed less than primary school, and illiteracy was more common compared to their urban counterparts. These factors may limit individuals’ ability to understand health information, communicate effectively with healthcare providers, and adhere to treatment regimens. Furthermore, a greater proportion of rural residents belonged to ethnic minority groups who may experience language barriers when accessing care. Together, these educational, linguistic, and communication barriers likely contribute to challenges in follow-up care and treatment adherence [32, 33], which may explain the lower hypertension control observed in rural settings. These rural–urban disparities in education and healthcare access may help explain the higher internal and doctor health locus of control scores observed among urban residents, and the higher chance locus of control scores among rural residents.

Among rural participants in this study, higher internal health locus of control scores—reflecting a stronger belief in one’s ability to influence personal health—were associated with greater odds of having controlled hypertension. However, internal health locus of control was not associated with awareness of hypertension or receipt of treatment. This finding suggests that internal health locus of control is positively correlated with better treatment outcomes, which is consistent with a previous study [34]. In contrast to findings from other settings, where internal health locus of control is often linked to information-seeking behaviors and increased health knowledge [35–37], this pattern was not observed in our rural sample. One possible explanation is the presence of structural barriers to healthcare access in rural Lao PDR, such as limited availability of health services for continued visits and medications availability. In such contexts, individuals may have a strong belief in personal responsibility for health but still face constraints in obtaining a diagnosis or accessing treatment. As a result, internal health locus of control may instead influence self-directed behaviors—such as adopting healthier diets or lifestyle changes—which can contribute to blood pressure control [4, 38] independent of formal healthcare engagement. On the other hand, among urban participants in this study—recruited from the capital city of Lao PDR—internal locus of control was not associated with hypertension awareness, receipt of treatment, or blood pressure control. Health locus of control is shaped by individuals’ lived experiences with health issues and is influenced by broader cultural and social contexts [12]. Research suggests that dominant health locus of control orientations may vary across cultural, age, and gender groups [39–41]. In urban settings, individuals may be more likely to rely on structured health systems and professional care, which could elevate the influence of doctor and other health locus of control subscales. In such contexts, people may place greater trust in healthcare providers to manage their care and feel less personally responsible for initiating or maintaining treatment—potentially diminishing the influence of internal locus of control on hypertension-related outcomes.

This study found that higher doctor and other people health locus of control scores were associated with greater hypertension awareness, treatment, and control among urban participants, but not among those living in rural areas. Previous research has shown that doctor and other people health locus of control is positively related to trust in the patient-physician relationship [42, 43]. In urban settings, where access to healthcare providers is more consistent and diverse, stronger beliefs in the influence of doctors and others may enhance patient trust and engagement with care [42, 44]. This trust may, in turn, support greater awareness of hypertension, increased likelihood of receiving treatment, and improved blood pressure control. In contrast, in rural settings, structural and contextual barriers may outweigh the influence of doctor and other people health locus of control. Factors such as low rates of health insurance coverage, long travel distances to healthcare facilities, lack of transportation, and restricted internet availability may limit the potential for doctor or other people health locus of control to positively affect health behaviors or outcomes in rural areas. Additionally, traditional health beliefs and practices may play a more prominent role in rural communities. For example, in a national survey, 12.1% of individuals aged 45 to 54 years reported currently taking herbal or traditional remedies for hypertension, and 26.7% had ever visited a traditional healer for this condition [10]. These practices may be even more common among rural populations, potentially diluting the influence doctor locus of control. This study also found that chance health locus of control was not associated with any of the hypertension outcomes among rural and urban participants. Although chance health locus of control is often negatively associated with healthy behaviors [44, 45], our finding was inconsistent with this pattern. Further studies, such as qualitative interviews, are needed to explore why chance health locus of control may be less relevant in this setting.

Our study has several limitations. First, blood pressure was evaluated at a single visit, whereas the use of averaged blood pressure obtained on two more occasions is recommended for accurate measurement [20]. However, trained personnel prepared the participants properly and used the proper technique to accurately measure blood pressure using a standardized protocol given limited resource setting. Second, self-reported awareness and treatment of hypertension were used as outcome measures. Self-reported data often overestimate medication adherence [46]. Therefore, the proportions of hypertension awareness and treatment in this study may have been overestimated. Future studies should consider using alternative or multiple measures, such as reviewing prescription records, to enhance the validity of these outcomes. Third, smoking status was based on self-reported lifetime cigarette consumption, which may be subject to recall bias. This should be considered when interpreting the results. Fourth, the measure of literacy applied in this study was based solely on the ability to read in the Lao language. Because the measure did not assess writing ability, it may not fully capture the broader concept of literacy and should be interpreted with caution. Furthermore, the results of the present study cannot be generalized to other settings such as communities with more limited resources. Even though the rural areas in this study were defined by the Lao Statistical Bureau, the rural areas where the participants were sampled are close to the capital city. The characteristics of the sample may be different from the characteristics of people in remote rural areas with more limited resources for healthcare service access. Lastly, p-values were not adjusted for multiple testing. To the best of our knowledge, this study is the first study to examine the association between hypertension awareness, treatment, and control and health locus of control in Lao PDR—a resource-limited setting. Although this study provides valuable insights into hypertension prevalence, its management, and the role of health locus of control, the number of statistical tests conducted increases the risk of Type I error, which may affect the interpretation of multiple associations. While the analytic sample size was relatively modest for subgroup analyses, a post hoc power analysis suggested that the sample may be sufficient to detect small effect sizes. Nonetheless, the findings should be interpreted with caution and considered exploratory.

## Conclusions

This study found a high prevalence of hypertension and low levels of awareness, treatment, and control among middle-aged adults in Lao PDR, with notable differences between rural and urban populations. Since the introduction of the National Health Insurance scheme in 2016, Lao PDR has made significant progress toward universal health coverage, with 94% of the population now enrolled in social protection programs [31]. Nonetheless, disparities in healthcare access related to socioeconomic status continue to exist [31, 47, 48]. The estimates from this study can serve as baseline data to assess how expanded healthcare access may influence hypertension outcomes in the years following the implementation of universal coverage.

Distinct patterns of health locus of control were associated with hypertension outcomes: Doctor and other people health locus of control were positively linked to awareness, treatment, and control in urban areas, while internal health locus of control was associated with control in rural areas. These findings highlight the need for context-specific interventions. In urban areas, strengthening trust in healthcare providers and leveraging social support systems may improve hypertension management. In rural areas, promoting self-management and culturally relevant education may be more effective, especially given access barriers.

## Supplementary Information

Supplementary Material 1.

Supplementary Material 2.

Supplementary Material 3.

## Data Availability

No datasets were generated or analysed during the current study.

## Abbreviations

Lao PDR: Lao People’s Democratic Republic
LMICs: Low- and middle-income countries
OR: Odds ratios
CI: Confidence intervals
SE: Standard error
HLOC: Health locus of control
