# Supplementary material for: Hypertension management and health belief in middle-aged laotian population: a cross-sectional survey
Source: BMC Public Health. 2025 Oct 3;25:3320. doi: 10.1186/s12889-025-24638-4 (PMC12495808; doi:10.1186/s12889-025-24638-4)
Supplement: Supplementary file 2 — Supplementary Material 2. [file 12889_2025_24638_MOESM2_ESM.docx]

**Table 1** Unweighted characteristics of participants by residential area

| Characteristics | *n (%)* | | |
| --- | --- | --- | --- |
|  | Rural  (*n* = 469) | Urban  (*n* = 453) | Total  (*N* = 922) |
| Age (in years) |  |  |  |
| 40-49 | 244 (52.0) | 237 (52.3) | 481 (52.2) |
| 50-59 | 225 (48.0) | 216 (47.7) | 441 (47.8) |
| Gender |  |  |  |
| Male | 232 (49.5) | 179 (39.5) | 411 (44.6) |
| Female | 237 (50.5) | 274 (60.5) | 511 (55.4) |
| Marital status |  |  |  |
| Never married | 6 (1.3) | 18 (4.0) | 24 (2.6) |
| Married or cohabiting | 406 (86.6) | 394 (87.0) | 800 (86.8) |
| Separated/divorced/widowed | 57 (12.2) | 41 (9.0) | 98 (10.6) |
| Ethnicity |  |  |  |
| Lao-Tai | 349 (74.4) | 449 (99.1) | 798 (86.6) |
| Other | 120 (25.6) | 4 (0.9) | 124 (13.4) |
| Employment status |  |  |  |
| Employed/paid job | 422 (90.0) | 338 (74.6) | 760 (82.4) |
| Not employed/non-paid job | 47 (10.0) | 115 (25.4) | 162 (17.6) |
| Educational attainment |  |  |  |
| No formal/less than primary | 130 (27.7) | 35 (7.7) | 165 (17.9) |
| Primary | 145 (30.9) | 87 (19.2) | 232 (25.2) |
| Secondary | 102 (21.8) | 101 (22.3) | 203 (22.0) |
| Tertiary or more | 92 (19.6) | 230 (50.8) | 322 (34.9) |
| Literacy |  |  |  |
| Yes | 383 (81.7) | 430 (94.9) | 813 (88.2) |
| No | 86 (18.3) | 23 (5.1) | 109 (11.8) |
| Health insurance |  |  |  |
| Yes | 78 (16.6) | 153 (33.8) | 231 (25.0) |
| No | 391 (83.4) | 300 (66.2) | 691 (75.0) |
| High-risk drinking |  |  |  |
| Yes | 245 (52.2) | 291 (64.2) | 536 (58.1) |
| No | 224 (47.8) | 162 (35.8) | 386 (41.9) |
| Smoker |  |  |  |
| Ever smoked | 137 (29.2) | 110 (24.3) | 247 (26.8) |
| Never smoked | 332 (70.8) | 343 (75.7) | 675 (73.2) |
| Health locus of control |  |  |  |
| Internal, mean (SD) | 27.6 (4.6) | 28.8 (5.2) | 28.2 (4.9) |
| Chance, mean (SD) | 24.6 (5.4) | 23.1 (6.5) | 23.8 (6.0) |
| Doctor, mean (SD) | 14.8 (2.1) | 15.4 (2.2) | 15.1 (2.2) |
| Other, mean (SD) | 13.1 (2.9) | 13.0 (3.3) | 13.0 (3.1) |

**Table 2** Weighted prevalence of hypertension, and proportions of awareness, treatment, and control by residential area

|  | *%* (*SE*) | | | |
| --- | --- | --- | --- | --- |
|  | Hypertension  (N = 922) | Awareness  (n = 441) | Treatment  (n= 441) | Control  (n = 441) |
| Overall | 44.3 (2.2) | 43.4 (3.1) | 42.3 (3.2) | 17.8 (2.2) |
| Residence |  |  |  |  |
| Urban | 46.0 (3.2) | 42.9 (4.3) | 41.6 (4.4) | 18.3 (2.8) |
| Rural | 41.6 (2.4) | 44.2 (4.1) | 43.7 (3.9) | 17.0 (3.4) |

SE, standard error.

Prevalence of hypertension was estimated from all participants (n = 922). Proportions of awareness, treatment, and control were estimated from participants who had hypertension (n = 441).

**Table 3** Weighted association between health locus of control and hypertension awareness, treatment, and control among urban participants (n = 230)

|  |  | *OR* (95% CI) | |  | |
| --- | --- | --- | --- | --- | --- |
|  | Awareness | | Treatment | | Control |
| Internal HLOC | 1.05 (1.00-1.11) | | 1.05 (1.00-1.10) | | 1.03 (0.96-1.11) |
| Chance HLOC | 1.03 (0.98-1.08) | | 1.03 (0.99-1.08) | | 1.01 (0.94-1.08) |
| Doctor HLOC | **1.15 (1.03-1.28)** | | **1.13 (1.01-1.27)** | | **1.25 (1.11-1.41)** |
| Other HLOC | **1.16 (1.06-1.27)** | | **1.14 (1.04-1.26)** | | **1.14 (1.02-1.28)** |

HLOC, health locus of control; OR, odds ratio; CI, confidence internal.

Separate models were fit for each combination of independent and dependent variables among individuals with hypertension living in urban areas. All models were adjusted for age, sex, educational attainment, employment status, ethnicity, and health insurance coverage. Estimates with p < 0.05 were presented in bold to indicate statistical significance. Analytic weights were applied to account for nonresponse and clustering of participants within villages.

**Table 4** Weighted association between health locus of control and hypertension awareness, treatment, and control among rural participants (n = 211)

|  |  | *OR* (95% CI) | |  | |
| --- | --- | --- | --- | --- | --- |
|  | Awareness | | Treatment | | Control |
| Internal HLOC | 1.06 (0.98-1.15) | | 1.06 (0.98-1.15) | | **1.17 (1.05-1.29)** |
| Chance HLOC | 1.05 (0.98-1.12) | | 1.04 (0.98-1.12) | | 1.09 (0.99-1.21) |
| Doctor HLOC | 1.14 (0.98-1.34) | | 1.15 (0.99-1.35) | | 1.07 (0.81-1.39) |
| Other HLOC | 1.10 (0.99-1.23) | | 1.10 (0.99-1.23) | | 1.22 (1.00-1.48) |

HLOC, health locus of control; OR, odds ratio; CI, confidence internal.

Separate models were fit for each combination of independent and dependent variables among individuals with hypertension living in rural areas. Each model was adjusted for age, sex, education attainment, employment status, ethnicity, and insurance. Estimates with p < 0.05 were presented in bold to indicate statistical significance. Analytic weights were applied to account for nonresponse and clustering of participants within villages.
